# Mapping strategies for reducing inequalities in adult elective surgical care in the United Kingdom: a living scoping review protocol

**DOI:** 10.1186/s13643-025-02927-8

**Published:** 2025-10-06

**Authors:** Katherine-Helen Hurndall, Jonathan Clarke, Ana-Luisa Neves, Ara Darzi

**Affiliations:** 1 Institute for Global Health Innovation, Imperial College London, St. Mary’s Hospital, Praed Street, London, United Kingdom; 2 The Health Foundation, London, United Kingdom

**Keywords:** Health inequalities, Elective surgery, Scoping review, United Kingdom, UK

## Abstract

**Background:**

Equitability of healthcare and timely access to services are essential components of healthcare quality, yet persistent health inequalities and inequity of access remain global challenges. The COVID-19 pandemic further exacerbated these disparities, underscoring the urgency to address health inequalities. In the United Kingdom (UK), despite longstanding recognition and efforts to tackle health inequalities, progress has been slow, with the pandemic highlighting stark disparities in healthcare outcomes and access. Renewed governmental initiatives and regional strategies aim to address these disparities, including within elective surgical care.

**Methods and analysis:**

This living scoping review protocol follows established methodological frameworks to map current evidence on strategies to reduce health inequalities in elective surgical care in the UK. Electronic databases (OVID Medline, Embase, Health Management Information Consortium, CINAHL) and grey literature sources will be systematically searched, with no time limit set, to identify relevant studies and documents. Selection criteria include articles describing strategies or policies aiming to reduce health inequalities in elective surgical care for adults in the UK, along with their impact on health outcomes. Data will be charted, collated, and summarised to provide a narrative description of findings.

**Discussion:**

This is the first living scoping review to map the available evidence on strategies to reduce inequalities in elective surgical care, across the whole patient pathway, in the UK. It is anticipated that this living review will be useful in informing future research, policy, and good practice. The findings of the scoping review will be submitted for peer-reviewed publication, presented at academic conferences, and discussed with a steering committee to inform future studies.

**Systematic review registration:**

The protocol was submitted to the Open Science Framework on 30/05/2024.

## Background

Equitability of healthcare and timely access to services are recognised by the Institute of Medicine and World Health Organisation (WHO) as key markers of healthcare quality [1, 2], yet health inequalities and inequality of access persist globally [3, 4]. “Reducing inequalities” achieving “gender equality” and promoting “good health and well-being” represent 3 of the 17 sustainable development goals set out in the WHO 2030 Agenda for Sustainable Development, adopted by all United Nations member states in 2015 [5]. The likeliness of achieving these goals, however, has been severely impacted by the COVID-19 pandemic, which revealed stark inequalities in healthcare and exposed the impact of the social determinants of health on overall health status.

In the United Kingdom (UK), health inequalities, defined as “avoidable and structural differences in health status and outcomes” [6] have been on the health and social care agenda for over 10 years. The startling reality of inequalities in health status and outcomes within the National Health Service (NHS) was showcased with the “Marmot review” [7] and the “Marmot review–10 years on” [8]. Despite this, progress to address these issues has been slow and the pandemic took advantage of such disparity, with black and ethnic minority groups, and those from a lower socioeconomic area, suffering higher COVID mortality and morbidity [9–12]. COVID-19 not only exposed inequality in health outcomes but showed inequality of access to care, with the most deprived UK communities experiencing maximal disruption to elective care during the pandemic and services in these areas being the slowest to recover [13, 14]. Following the pandemic, there is renewed appetite to address health inequalities at all levels. At governmental level, the 2022 Health and Social Care Act described the biggest NHS reforms in years with a clear focus on reducing bureaucracy, restructuring and “joining-up” care and services, to reduce health inequalities [15]. Similarly, the 2019 “NHS Long Term Plan” published by NHS England described key actions the health service should undertake to reduce inequalities [16]. In 2021, “The National Healthcare Inequalities Improvement Programme” was created to work across policies and programmes to deliver high-quality, equitable care. The “CORE20PLUS5” initiative aims to improve outcomes and access to care for the most deprived 20% of the UK population (plus underserved population groups identified at local level) across five clinical areas: maternity; severe mental illness; chronic respiratory disease; early cancer diagnosis, and hypertension and lipid optimisation [17]. Regionally, healthcare services are co-ordinated by Integrated Care Boards (ICB), each dedicated to serving four core purposes, including “tackling inequalities in outcomes, experience and access” [18]. Some ICBs, for example Dorset and Coventry & Warwickshire, have created committees dedicated to reviewing health inequalities and tackling variations in outcomes [19, 20]. Others, for example North East London and Hampshire and Isle of Wight ICBs, have developed strategic plans to fully encapsulate their local health inequality agendas across all aspects of improvement programmes [21, 22]. Despite some areas of good practice, no ICBs reported feeling “very confident” in their ability to deliver on health inequalities in a 2022/23 survey [23], prompting the NHS Confederation to recently publish a practical guide on embedding action on health inequalities in Integrated Care Systems [18].

For elective surgical care, NHS England instructed hospitals to disaggregate their elective waiting lists by ethnicity and deprivation, prioritising service delivery with this in consideration to recover elective services inclusively [24]. The policy was broad, with different trusts implementing a variety of strategies ranging from using artificial intelligence for waiting list prioritisation to targeted prehabilitation to optimise patients for surgery [25, 26]. Despite stated policy, however, there is no evidence of dedicated approaches to tackle  inequalities in elective surgery across the NHS [24] and there is limited evidence of the success of these strategies in reducing inequalities in care [24]. This living scoping review will map the current evidence on strategies to reduce inequalities in elective surgical care in the UK across the patient pathway. Where available, the review will report the effects of interventions on key patient outcomes, including mortality, morbidity, waiting times, and rates of hospital reattendances or readmissions. These outcomes are widely recognised indicators of healthcare quality and are routinely captured within UK hospital administrative datasets. Consequently, it is anticipated that many included studies will assess the impact of their interventions using these measures.

## Methods

Scoping reviews allow the exploration of broad topics with a heterogenous subject base to allow researchers to identify key concepts and gaps in knowledge [27]. As such, this study is best suited to this methodology to allow the published literature to be mapped in a comprehensive way to guide future research in this field. Living reviews are a method of addressing the evidence-practice gap and are suited for studies in which evidence is currently limited or changing at a fast pace [28]. As policies take time to be implemented and take effect, publishing this work as a living scoping review is ideal to facilitate up-to-date publications without creating research waste [28].

**Fig. 1:**
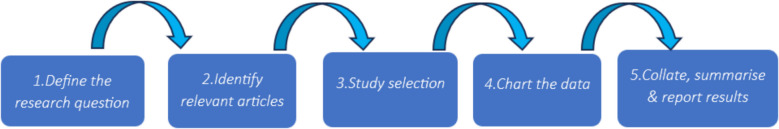
Methodological framework for scoping reviews

This scoping review protocol follows the methodological framework defined by Arksey and O’Malley [29] and enhanced by Levac et al. [30] (Fig. 1 Methodological Framework for Scoping Reviews). A 6th stage, consultation exercise, is recommended; however, due to time constraints, it will not be included in this scoping review. The scoping review is registered with the Open Science Framework, and the subsequent manuscript will follow the reporting guidelines from the Preferred Reporting Items for Systematic Reviews and Meta-Analyses (PRISMA) scoping review extension [31]. The protocol for this review was developed considering the PRISMA-P 2015 checklist (Supplementary file 1). The Cochrane guidance for the production and publication of living systematic reviews [32] will be used to produce the living scoping review.

### Step 1: identifying the research questions

Prior to defining the research questions, a preliminary literature review was conducted to understand the literature available on strategies to reduce inequalities in elective surgical care. This phase primarily showed literature focusing on strategies to reduce waiting times for elective surgery and inequalities within the waiting list. This aligns with waiting times being a commonly measured metric in many countries, particularly those with publicly funded healthcare services, used to evaluate the performance of the health service [33]. This preliminary search informed the decision to identify strategies and policies aimed at reducing inequalities in elective surgery more broadly and to describe their impact on health outcomes. Based on this, the following research questions have been identified:

1. What are the current interventions or policies to reduce inequalities across the adult elective surgical care pathway in the UK?
2. How many of these studies report patient outcomes from their intervention?
3. Of the studies which report patient outcomes, which outcomes are measured and what is the effect of the intervention on these outcomes?
4. Do any studies evaluate the impact of the intervention on the non-targeted population? If so, what was their effect?

### Stage 2: identifying relevant studies

Electronic searches will be conducted using OVID MEDLINE, Embase, Health Management Information Consortium (HMIC) and CINAHL. These databases have been selected as they are a comprehensive source of health literature, including literature on health service organisation and policy. A variety of grey literature will also be searched through agency websites such as the Department of Health and Social Care, the King’s Fund library, NHS England’s Knowledge and Library Hub and Google Scholar to identify studies, policy documents and reports relevant to the research questions. Supplementary articles may be obtained from reference searching of relevant articles. A full search strategy for OVID Medline, Embase, HMIC and CINAHL was developed following guidance from a subject librarian using a combination of keywords and Medical Subject Headings (MeSH) terms (Table 1). The search strategy will be adapted for each electronic database and grey literature information source.

**Table 1:** Full search strategy in OVID MEDLINE, conducted April 2024

#	Search	Results
1	(inequit* OR inequalit* OR disparit*).mp [mp = title, book title, abstract, original title, name of substance word, subject heading word, floating sub-heading word, keyword heading word, organism supplementary concept word, protocol supplementary concept word, rare disease supplementary concept word, unique identifier, synonyms, population supplementary concept word, anatomy supplementary concept word]	194,397
2	Health inequalities.mp. [mp = title, book title, abstract, original title, name of substance word, subject heading word, floating sub-heading word, keyword heading word, organism supplementary concept word, protocol supplementary concept word, rare disease supplementary concept word, unique identifier, synonyms, population supplementary concept word, anatomy supplementary concept word]	8034
3	Exp Health Inequities/	41,149
4	(strateg* OR polic* OR intervention*).mp. [mp = title, book title, abstract, original title, name of substance word, subject heading word, floating sub-heading word, keyword heading word, organism supplementary concept word, protocol supplementary concept word, rare disease supplementary concept word, unique identifier, synonyms, population supplementary concept word, anatomy supplementary concept word]	3,309,779
5	Health policy/or health care reform/	103,227
6	(elective OR planned OR non-urgent).mp. [mp = title, book title, abstract, original title, name of substance word, subject heading word, floating sub-heading word, keyword heading word, organism supplementary concept word, protocol supplementary concept word, rare disease supplementary concept word, unique identifier, synonyms, population supplementary concept word, anatomy supplementary concept word]	208,426
7	1 OR 2 OR 3	194,831
8	4 OR 5	3,334,637
9	6 AND 7 AND 8	804
10	(“United Kingdom OR “England” OR “UK”).mp. [mp = title, book title, abstract, original title, name of substance word, subject heading word, floating sub-heading word, keyword heading word, organism supplementary concept word, protocol supplementary concept word, rare disease supplementary concept word, unique identifier, synonyms, population supplementary concept word, anatomy supplementary concept word]	477,181
11	(“National Health Service” OR “NHS”).mp. [mp = title, book title, abstract, original title, name of substance word, subject heading word, floating sub-heading word, keyword heading word, organism supplementary concept word, protocol supplementary concept word, rare disease supplementary concept word, unique identifier, synonyms, population supplementary concept word, anatomy supplementary concept word]	52,656
12	10 OR 11	499,499
13	9 AND 12	88

To ensure a breadth of literature is returned, no time limit has been set to the search criteria. All identified literature will be imported into Covidence, a web-based systematic review platform [34], and duplicates will be removed.

The search will be repeated every six months, and any relevant new evidence will be used to update the review.

### Stage 3: study selection

#### Selection process

The review will be conducted in Covidence [34] and articles will be systematically screened according to a three-stage process. In stage 1, the title and abstract of all returned studies will be screened by the first reviewer. In stage 2, two reviewers will independently screen the full texts and apply the eligibility criteria below. In stage 3, if the independent reviewers cannot agree on the eligibility of an article, it will be referred to a third independent reviewer and will be included according to majority consensus. A PRISMA flow diagram [35] will be included to show the number of included and excluded studies, along with the rationale for exclusion. At six monthly intervals, the first reviewer will re-run the search and independently review any new articles for inclusion in subsequent updates.

#### Eligibility criteria

##### Definitions

Adults are defined as those persons aged 18 years or older. Elective surgery is defined as a non-urgent, planned operation or procedure.

#### Inclusion criteria

##### Population

Adults who have received or are waiting for elective surgery in the UK.

##### Concept

- Articles describing initiatives or policies at national, regional, or local level which aim or have been shown to reduce inequalities.
- Articles describing the effect of strategies to reduce inequalities on one or more of the following health outcomes:
  1. Mortality
  2. Morbidity
  3. Emergency reattendances or readmissions
  4. Waiting times
- Articles describing the effect of strategies to reduce inequalities on the non-target population

##### Context

- Elective surgical care
- UK

##### Type of study

Studies written in English.

#### Exclusion criteria

- Strategies focused on emergency care.
- Papers focusing on paediatric services. Paediatric services are defined as any service which cares for patients below the age of 18 years.
- Non-English language publications.
- Books.
- Qualitative research.

Qualitative research will be excluded as they are specific to the participants studied and are unlikely to describe the outcomes of interest, which were selected to reflect commonly used measures of care quality.

### Stage 4: charting the data

A data extraction form has been created, and the charting domains and subdomains are described in Table 2. Data charting will be conducted in Covidence by two independent reviewers. To ensure understanding and correct usage of the data extraction tool, 10% of articles will be charted simultaneously by the two independent reviewers, and the findings compared. After this, the remaining articles will be reviewed independently. If there are discrepancies between the two reviewers which cannot be resolved, a third reviewer will be consulted to reach consensus. The draft extraction form will be modified, if necessary, during the extraction process, and modifications will be detailed and justified in the completed scoping review manuscript.

**Table 2:** Data extraction form

Category	Data type
Article Information	1. Article title 2. Authors 3. Publication year 4. Publication type 5. Journal or place of publication 6. Population studied and size
Research questions	1) Summary of intervention or policy reported 2) How many studies demonstrate an effect on patient outcomes? 3) What is the impact of the intervention on i) Mortality ii) Morbidity or Post-operative complications iii) Overall waiting time iv) Emergency reattendance readmission 4) How many studies evaluate the impact of the intervention on the non-targeted population? 5) Of the studies which evaluate the above, what is the described impact?

### Stage 5: collating, summarising, and reporting the results

Data will be collated and summarised to produce a narrative description of study findings, with study characteristics presented graphically or in tables, where appropriate. Qualitative analysis of the included interventions will also be performed, with the aim of identifying key themes of intervention, with mapping of these across the surgical patient pathway.

### Patient and public involvement

Whilst patients and the public have not been involved in the creation of this study protocol, a workshop with a steering committee comprising health professionals and patients/public members will be conducted to discuss the results of the study. This will help frame the findings of the scoping review and further identify gaps in the research to explore.

## Discussion

This living scoping review will be the first to identify strategies employed at national, regional, and local levels to address inequalities in elective surgery and map these across the elective surgical care pathway to characterise themes of intervention. It is expected that the review will identify areas of good practice, allowing UK healthcare providers, policy makers, and health professionals to inform practice. We hypothesise, however, that there will be a lack of evidence describing the impact of these strategies on health outcomes for both the targeted and non-targeted populations; therefore, identifying key areas for future research.

## Data Availability

The datasets used and/or analysed during the current study are available from the corresponding author on reasonable request.

## Abbreviations

WHO: World Health Organisation
UK: United Kingdom
NHS: National Health Service
ICB: Integrated Care Board
PRISMA: Preferred Reporting Items for Systematic Reviews and Meta-Analyses
HMIC: Health Management Information Consortium
MeSH: Medical Subject Headings
